# Draft genome of a member of ascomycotal fungal genus *Pseudopithomyces* (family Didymosphaeriaceae)

**DOI:** 10.1128/mra.00173-23

**Published:** 2025-11-25

**Authors:** Krithika Arumugam, Sherilyn Ho, Irina Bessarab, Falicia Q. Y. Goh, Mindia A. S. Haryono, Ezequiel Santillan, Stefan Wuertz, Yvonne Chow, Rohan B. H. Williams

**Affiliations:** 1 Singapore Centre for Environmental Life Sciences Engineering (SCELSE), Nanyang Technological University 54761, Singapore, Singapore; 2 Singapore Institute of Food and Biotechnology Innovation (SIFBI), Agency for Science, Technology and Research (A*STAR) 54759 , Singapore, Singapore; 3 Singapore Centre for Environmental Life Sciences Engineering (SCELSE), National University of Singapore 37580, Singapore, Singapore; 4 School of Civil and Environmental Engineering, Nanyang Technological University 54761, Singapore, Singapore; University of California Riverside, Riverside, California, USA

**Keywords:** nanopore sequencing, ascomycota, pleosporales, didymosphaeriaceae, wastewater, fungal genomics

## Abstract

We report a draft genome of a species from the ascomycotal fungal genus *Pseudopithomyces* (isolate name SBW1) obtained using a culture isolate from brewery wastewater. The 43 contig genome has a length of 39.65 Mbp, with Benchmarking Universal Single-Copy Orthologs (BUSCO) completeness of 98.9%. Taxonomic assignment was supported by internal transcribed spacer (ITS) sequence, mitochondrial DNA sequence, and *k*-mer analysis.

## ANNOUNCEMENT

*Pseudopithomyces* (1) is a fungal genus whose species are implicated as human and animal pathogens (2–4). One draft genome (3) of *Pseudopithomyces chartarum* is publicly available (GenBank GCA_033220655.1; see also (5) for emerging genome data from this genus). We report a genome of a species of *Pseudopithomyces* isolated from brewery wastewater in Singapore. An earlier version of this genome (6), built using long-read data, was annotated as *Pseudopithomyces. maydicus* strain SBW1 (GenBank GCA_026873275.1).

We obtained an isolate by culturing on solid Yeast Extract–Peptone–Dextrose agar media at 30°C for 2 days. Genomic DNA was extracted using liquid nitrogen and mechanical grinding followed by application of the Qiagen DNeasy PowerSoil Pro Kit. Initial taxonomic classification was made via Sanger sequencing of the D1/D2 domain of the 28S SSU-rRNA gene, amplified using the NL1 and NL4 primer pair, and annotated, via the NCBI BLASTN webserver (https://blast.ncbi.nlm.nih.gov/Blast.cgi; 7) against the nr/nt database, to *P. maydicus* (MF919633) with 99% percent identity (PID) and 66% query coverage (qcovs).

Sequencing libraries were prepared using the SQK-LSK109 Ligation Sequencing kit (Oxford Nanopore Technologies, ONT), barcoded using the EXP-NDB104 Native Barcoding kit, and sequenced on a GridION (ONT; release 21/05/20) for 72 hours. An Illumina TruSeq Nano library was constructed and sequenced on an Illumina MiSeq (301 bp paired-end).

**Fig 1:**
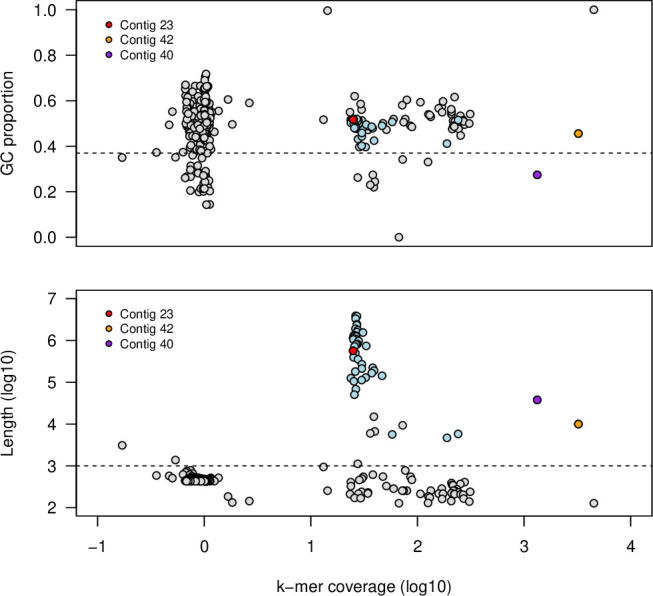
Recovery of draft genome from all contigs in the total assembly, showing *A*) plot of contig (*k*-mer) coverage (log10) versus contig GC proportion, and *B*) plot of contig (*k*-mer) coverage (log10) versus contig length (log10). Data points colored blue are contigs in the draft genome. Selected contigs are highlighted: contig 23 and contig 42 contain rRNA operons, including full-length ITS regions; contig 40 is annotated to the mitochondrial genome. Gray points show contigs that are not included in the draft genome; these are either short (<1000 bp) or hold GC values <0.37, which was the limit of the GC in the main group of long contigs. The cluster of short contigs with very low abundance likely represents sequence from the kitome or other contaminant sources, and the set of short (<1 kbp) with higher abundances is likely mis-assembled led fragments from the chromosomal, mitochondrial, or non-chomosomal sequences.

In total, 1.53 Gbp of basecalled and filtered (https://github.com/rrwick/Porechop) GridION reads (mean length: 6,717 bp) and 3.28 Gbp (*n* = 5,455,152) of paired-end Illumina reads were obtained and assembled using hybridSPAdes (version 3.15.5-Linux; flags –isolate, --nanopore, -t 30 and -k 21,33,55,77,99,127) (8). From 418 contigs (see Additional File 1 at https://zenodo.org/records/15668185), a draft genome of 43 contigs was recovered (Fig. 1; further details in legend; see Additional File 2 at https://zenodo.org/records/15668185), with a GC content of 49.9%, median *k*-mer coverage (*C_k_*) of 27, total length of 39,656,604 bp (N50: 1.76 Mbp) and BUSCO (9) completeness of 98.9% (BUSCO: C:98.9% [S:98.9%, D:0.0%], F:0.5%, M:0.6%, n:758; tested against the fungal_odb10 lineage data set; BUSCO v 5.4.7).

Two full-length ITS regions were identified using ITSx (10), one from the main ensemble of contigs (contig 23; Fig. 1) and the second (contig 42; Fig. 1) with very high coverage (*C_k_* = 3,230), suggesting a high (3,230/27 ≈120) rRNA copy number (11). ITS sequences (see Additional File 3 at https://zenodo.org/records/15668185) were annotated using NCBI BLASTN against the NCBI rRNA/ITS database (https://www.ncbi.nlm.nih.gov/bioproject/177353). The first ITS region annotated (top five hits; see Additional File 4 at https://zenodo.org/records/15668185) to *Paraphaeosphaeria* and *Pseudopithomyces*, but with 61% qcovs and 72–73% PID, suggesting poor quality reconstruction. From the second ITS region (see Additional File 5 at https://zenodo.org/records/15668185), the top three hits were to *Pseudopithomyces* (qcovs: 87–100%; PID: 96–97%), and the fourth and fifth hits were *Paraconiothyrium brasiliense* and *Paracamarosporium hawaiiense*, respectively, but with far lower specificity (qcovs: 84–88%; PID: 94–95%).

A third high abundance contig (contig 40; 37,881 bp, *C_k_* = 1,328; Fig. 1) was annotated to the mitochondrial genome of *P. chartarum* (NC_035636.1; qcovs: 81%; PID: 97%; Additional File 6 at https://zenodo.org/records/15668185). Whole-genome analysis using sourmash (12) against 18,883 NCBI fungal genomes returned *P. chartarum* (GCA_033220655.1) as the closest hit (see Additional File 7 at https://zenodo.org/records/15668185).

Collectively, these findings support a taxonomic annotation to a species of *Pseudopithomyces*, designated as *Pseudopithomyces* sp. SBW1.

## Data Availability

Additional data files listed above are available at the following Zenodo repository: https://zenodo.org/records/15668185, including FASTA files for the entire assembly and for the draft genome. The draft genome sequence is being deposited at DDBJ/ENA/GenBank to replace our previous submission JANTUC000000000 originally annotated as Pseudopithomyces maydicus strain SBW1. FASTQ data used in this paper is available at European Nucleotide Archive (ENA) via Biosample accession SAMEA118333352 (Study accession PRJEB89409).
